# Feasibility and Acceptability of a Nurse-Led Active Music Program for Older Adults with Cognitive Impairment in Long-Term Care: A Pilot Quasi-Experimental Study

**DOI:** 10.3390/healthcare14213732

**Published:** 2026-11-03

**Authors:** Ana Belén Villegas-Aparicio, Agustín J. Simonelli-Muñoz, Diana Jiménez-Rodríguez, Fernando Plaza del Pino, Juana Inés Gallego-Gómez

**Affiliations:** 1 Virgen del Rosario Long-Term Care Residence, Avda. Juan Carlos I, 246, 04740 Roquetas de Mar, Almería, Spain; 2 Department of Nursing, Physiotherapy and Medicine, University of Almería, 04120 Almería, Spain

**Keywords:** acceptability, cognitive impairment, feasibility, long-term care, music-based intervention, non-pharmacological intervention, nursing, older adults, person-centered care, vulnerable populations

## Abstract

**Background/Objectives:** Older adults with cognitive impairment living in long-term care facilities are a vulnerable population with complex cognitive, emotional, and social care needs. This pilot study evaluated the feasibility and acceptability of a structured, nurse-led active music program and explored preliminary changes in cognitive function, depressive symptoms, and session-based engagement. **Methods:** A single-group quasi-experimental pre–post study was conducted in one long-term care facility. Eleven residents were enrolled, and nine completed an eight-session, four-week group intervention led by a registered nurse. Feasibility was assessed through retention, attendance, and intervention delivery, and acceptability through satisfaction and willingness to participate again. Cognitive function and depressive symptoms were assessed using the Montreal Cognitive Assessment (MoCA) and the 15-item Geriatric Depression Scale (GDS-15), respectively. Participation, sustained attention, and social interaction were recorded during each session. Pre–post changes were analyzed using exact Wilcoxon signed-rank tests. **Results:** Retention was 81.8%. Among participants who completed the intervention, attendance was 100% (72/72 scheduled participant-sessions). Satisfaction was 5.00 ± 0.00, and all participants reported that they would be willing to participate again. Engagement scores were moderate to high across sessions. MoCA scores increased from 22.89 ± 2.20 to 23.89 ± 2.03 (*p* = 0.016), whereas GDS-15 scores showed no significant change (6.33 ± 1.00 vs. 6.00 ± 1.00; *p* = 0.250). **Conclusions:** The nurse-led active music program showed favorable feasibility and acceptability indicators in this long-term care setting. Preliminary findings regarding cognitive function warrant further investigation in larger controlled studies, whereas no measurable change in depressive symptoms was observed.

## 1. Introduction

Population aging is increasing the demand for geriatric and long-term care services worldwide. In Spain, adults aged 65 years and older accounted for 21.1% of the population in 2026, and this proportion is projected to reach 30.9% by 2076, according to the Spanish National Statistics Institute (INE) [1].

Although pharmacological treatment remains necessary for specific clinical conditions, medication use in older adults living in long-term care facilities requires particular caution, especially when psychotropic drugs are used to manage behavioral and psychological symptoms associated with cognitive impairment and dementia. In this population, psychotropic drug use has been associated with adverse outcomes, including falls and fractures, cardiovascular and cerebrovascular events, and increased mortality [2]. Consequently, there is growing interest in safe, feasible, and person-centered non-pharmacological interventions that can be incorporated into routine long-term care practice [2,3].

Music-based interventions have emerged as a promising non-pharmacological approach for older adults with cognitive impairment. Evidence from systematic reviews and clinical studies suggests potential benefits for cognitive functioning, depressive and anxiety symptoms, emotional well-being, and social connectedness, although findings remain heterogeneous according to intervention characteristics and study design [4,5,6,7,8,9]. Active music interventions may be particularly relevant in residential care because they extend beyond passive listening and encourage direct involvement through singing, rhythmic movement, melody recognition, autobiographical reminiscence, and sustained attention. These activities may promote behavioral engagement, emotional expression, and interpersonal interaction in settings where opportunities for structured and meaningful activity may otherwise be limited [7,8].

An important consideration for healthcare services is not only whether such interventions may produce beneficial outcomes but also whether they can be delivered feasibly within existing care structures. Nurse-led interventions may be particularly relevant in long-term care settings because nurses are well positioned to deliver structured non-pharmacological interventions as part of routine care, potentially offering advantages in terms of resource use, patient safety, continuity of care, and satisfaction [10]. Integrating structured non-pharmacological programs into routine nursing practice may also facilitate their incorporation into existing care structures. In this context, active music programs can be conceptualized not merely as recreational activities but as structured psychosocial nursing interventions that contribute to person-centered care and therapeutic engagement [10,11,12].

Standardized nursing terminologies may further support the systematic integration of these interventions into clinical practice. NANDA-I nursing diagnoses can be used to identify relevant human responses associated with cognitive impairment, loneliness, and social isolation, while the Nursing Interventions Classification (NIC) provides a framework for interventions such as Music Therapy, Cognitive Stimulation, Reminiscence, Active Listening, and Socialization Enhancement [13,14]. Nursing Outcomes Classification (NOC) outcomes can, in turn, support the identification of expected cognitive, emotional, and social dimensions of care. The integration of NANDA-I, NIC, and NOC may therefore provide a structured clinical reasoning framework for the planning and delivery of person-centered nursing interventions [15,16].

The terms NANDA-I, NIC, and NOC refer to established nursing classification systems [15,16]; use of the NIC label “Music Therapy” does not indicate that a credentialed music therapist delivered this intervention.

Despite increasing evidence on music-based interventions for cognitive impairment and dementia, substantial heterogeneity remains regarding intervention content, the professionals responsible for delivery, and the organizational conditions required for implementation in routine long-term care. Recent research has highlighted the importance of feasibility, acceptability, adherence, organizational barriers, and sustainability when translating music-based interventions from research settings into everyday care [17,18,19]. However, evidence remains limited regarding structured active music programs delivered directly by nursing staff within long-term care facilities where residents receive their usual care. Establishing feasibility and acceptability is therefore an important preliminary step before larger controlled effectiveness studies are undertaken.

Accordingly, the aim of this pilot study was to evaluate the feasibility and acceptability of a structured nurse-led active music program for older adults with cognitive impairment living in a long-term care facility. As exploratory outcomes, the study also examined pre–post changes in cognitive screening performance and depressive symptoms, together with session-based levels of active participation, sustained attention, and social interaction. Given the pilot design and small sample size, clinical outcomes were considered preliminary and were not intended to establish intervention efficacy.

## 2. Materials and Methods

### 2.1. Study Design and Setting

This pilot quasi-experimental study used a single-group pre–post design and was designed as a proof-of-concept study to examine the feasibility, acceptability, and preliminary clinical effects of a structured non-pharmacological intervention delivered in a real-world long-term care setting. Similar exploratory intervention designs have been used to investigate music-based approaches in older adults with cognitive impairment and dementia [20,21]. Given the absence of a parallel control group, the study was not designed to establish causal relationships between the intervention and the observed outcomes.

The study was conducted at the Virgen del Rosario Long-Term Care Residence in Roquetas de Mar, Almería, Spain, a privately managed, publicly subsidized long-term care facility providing care to older adults with varying degrees of functional dependency and neurodegenerative conditions. Intervention sessions were conducted in a familiar and accessible room within the facility. The environment was arranged to minimize distracting stimuli and promote orientation, comfort, and participation.

### 2.2. Participants and Recruitment

Residents meeting the predefined eligibility criteria were recruited consecutively from the long-term care facility. Eleven residents were initially enrolled in the study. Two participants discontinued the program before the second session, resulting in a final sample of nine participants who completed the intervention and both pre- and post-intervention assessments.

The facility psychologist had documented mild cognitive impairment before recruitment, independently of the MoCA screening administered for the study. All eleven enrolled participants were coded as having mild cognitive impairment in the baseline dataset; diagnostic criteria beyond the existing clinical documentation were not independently verified for this study.

Eligible participants were aged 70 years or older, had been permanently residing in the facility for at least one month, and had a documented clinical diagnosis or screening result consistent with mild-to-moderate cognitive impairment. Participants were also required to have sufficient motor capacity to participate in seated group activities and adequate or corrected hearing ability to understand verbal instructions and engage in the musical activities.

Exclusion criteria were severe uncorrected hearing impairment; an unstable medical condition, decompensated disease, or terminal illness that could compromise safe participation; severe uncontrolled neuropsychiatric symptoms, including delusions, hallucinations, or marked agitation; and refusal to participate.

As this was a pilot feasibility study, no formal a priori sample size calculation based on statistical power was performed. The primary purpose was to determine whether the intervention could be implemented and tolerated in routine long-term care practice and to obtain preliminary information to inform the design of future controlled studies, consistent with methodological recommendations for feasibility pilot studies [22].

No prospectively defined feasibility or acceptability success thresholds or progression criteria were established.

### 2.3. Intervention

The intervention consisted of a structured, group-based active music program led by a registered nurse employed at the long-term care facility. The program was delivered over four consecutive weeks, with two sessions per week, for a total of eight sessions. Each session lasted approximately 40 min.

The same nurse facilitated all sessions. As a member of the facility staff, she was familiar with the participants’ clinical characteristics, functional limitations, and personal preferences, allowing activities to be adapted to individual capabilities while continuously monitoring safety and tolerance. This individualized approach was consistent with principles of person-centered care for people living with cognitive impairment in residential settings [23].

The facilitator had no specialist training in music therapy or formal music education. A written eight-session protocol existed before implementation (Supplementary Material S1). The same nurse delivered all sessions during her regular working hours; nursing workload was not formally measured.

Participants were seated in a semicircle to facilitate visual contact and group interaction. Materials included an audio speaker, large-print song sheets, and simple percussion instruments, such as maracas and tambourines. The musical repertoire was selected according to participants’ biographical and musical preferences and primarily comprised familiar Spanish popular and traditional songs from the 1950s to the 1970s to promote autobiographical reminiscence and active engagement. Previous research in nursing home residents with cognitive impairment has similarly supported the use of preferred and personalized music interventions [24].

Each session followed a standardized three-phase structure. The orientation phase included brief temporal orientation, a personalized rhythmic greeting, and simple breathing exercises. The active stimulation phase comprised group singing, recognition and completion of familiar songs, call-and-response singing, body percussion, the use of simple percussion instruments, and coordinated seated movements. The closing phase included relaxation with soft instrumental music and an opportunity for participants to verbalize memories, emotions, or impressions evoked during the session.

Throughout each session, the nurse adjusted the pace and intensity of activities according to participants’ fatigue and responsiveness, controlled the sound volume to prevent overstimulation, and supervised participant safety. Observational data were recorded after each session.

Following each session, the facilitator qualitatively recorded the activities delivered. According to her confirmation and records, no changes to the planned activities were required. These records were not a formal, standardized fidelity checklist, and no independent fidelity observer was employed.

The intervention was structured using a nursing clinical reasoning framework based on NANDA-I diagnoses, the Nursing Interventions Classification (NIC), and the Nursing Outcomes Classification (NOC) [25,26,27]. Relevant nursing interventions included Music Therapy, Cognitive Stimulation, Reminiscence, Active Listening, and Socialization Enhancement. NOC outcomes were used to guide the expected cognitive, emotional, and social dimensions of care but were not used as direct research outcome measures. The use of this framework was intended to support a systematic and person-centered approach to intervention planning and delivery [23].

This structured nursing intervention was not specialist-delivered music therapy; “Music Therapy” is used only when naming the corresponding NIC intervention.

### 2.4. Outcome Measures

Feasibility and acceptability constituted the primary domains of interest, whereas cognitive function and depressive symptoms were considered exploratory clinical outcomes.

#### 2.4.1. Feasibility and Acceptability Measures

Feasibility was assessed through participant retention, intervention completion, attendance at scheduled sessions, and successful delivery of the eight-session program within the long-term care setting.

Acceptability was measured in completers only. Feasibility and acceptability outcomes were described without comparison with prospectively specified benchmarks.

Acceptability was assessed after completion of the intervention using an ad hoc questionnaire (Supplementary Material S2). Participants rated their overall satisfaction with the program on a 5-point Likert scale ranging from 1 (completely dissatisfied) to 5 (completely satisfied) and indicated whether they would be willing to participate in the program again.

#### 2.4.2. Assessment of Cognitive Function

Cognitive function was assessed using the Spanish version of the Montreal Cognitive Assessment (MoCA) [28,29]. The MoCA is a cognitive screening instrument with a total score ranging from 0 to 30, with higher scores indicating better global cognitive performance. It assesses several cognitive domains, including attention, short-term memory, language, executive functioning, visuospatial abilities, abstraction, and orientation. The official Spanish-language MoCA form was used, with its education correction applied according to the instrument instructions. The scores recorded in the database already incorporated this adjustment; no additional education correction was applied during analysis.

The MoCA was administered individually during the week preceding the intervention (T0) and again during the week following completion of the eighth session (T1).

The MoCA was administered individually by the same nurse who delivered the program; assessor blinding was not possible.

#### 2.4.3. Assessment of Depressive Symptoms

Depressive symptoms were assessed using the 15-item Geriatric Depression Scale (GDS-15) [30,31]. The instrument comprises 15 dichotomous items and yields a total score ranging from 0 to 15, with higher scores indicating a greater burden of depressive symptoms.

The GDS-15 was administered individually at baseline (T0) and after completion of the intervention (T1).

Questions were read aloud individually by the nurse, who recorded participants’ answers. The same nurse administered both baseline and post-intervention assessments and was not blinded to time point.

#### 2.4.4. Session-Based Observational Measures

Behavioral engagement was assessed during each of the eight sessions using a structured observational record developed for the study. Three prespecified dimensions were analyzed: active participation, sustained attention, and social interaction. Each dimension was rated on a 5-point ordinal scale, with higher scores indicating greater engagement.

Active participation reflected involvement in singing, rhythmic, and movement activities. Sustained attention reflected the ability to remain engaged with the facilitator’s instructions and session activities. Social interaction reflected verbal or non-verbal communication with other participants or with the facilitator.

The five-point observational record was completed by the nurse within 15 min after each session. The original recording fields and the absence of prospectively documented scoring anchors are described in Supplementary Material S3; no independent reliability testing or formal assessor training was performed.

For each participant, mean scores across attended sessions were calculated for the three dimensions.

### 2.5. Data Management and Statistical Analysis

Participant data were entered into a coded database using unique study identifiers. Baseline characteristics, session-based observational data, and pre- and post-intervention assessments were linked using the participant identifier. No missing values were imputed. Pre–post analyses were based on complete paired observations from the nine participants who completed both assessments.

Descriptive statistics were used to characterize the sample and summarize feasibility, acceptability, and clinical outcomes. Categorical variables were expressed as frequencies and percentages. Continuous variables were summarized using means and standard deviations (SDs) and, where appropriate, medians and interquartile ranges (IQRs). Session-based participation, sustained attention, and social interaction scores were summarized across the intervention sessions for each participant.

For descriptive comparisons of completers and non-completers, baseline characteristics were summarized without inferential tests. Sample quartiles of paired changes were calculated using linear interpolation (25th/75th percentiles).

Given the small sample size, the distribution of paired pre–post differences was examined using the Shapiro–Wilk test. Because the normality assumption was not adequately satisfied, changes in MoCA and GDS-15 scores between baseline (T0) and post-intervention (T1) were analyzed using the Wilcoxon signed-rank test for paired observations. Two-sided exact *p*-values were used, with statistical significance set at *p* < 0.05.

Given the pilot nature of the study, analyses of cognitive function and depressive symptoms were considered exploratory. Statistical significance was therefore interpreted cautiously and was not considered evidence of intervention efficacy in the absence of a control group.

Exploratory paired mean differences are additionally described using two-sided 95% confidence intervals (CIs) calculated from the paired t distribution and Cohen’s dz (paired mean difference divided by its standard deviation). These intervals and standardized values do not establish causal efficacy.

Statistical analyses were performed using IBM SPSS Statistics, version 31.0 (IBM Corp., Armonk, NY, USA).

### 2.6. Ethical Considerations

The study was conducted in accordance with the principles of the Declaration of Helsinki and applicable Spanish and European regulations governing health and social care research. The study protocol and data collection procedures were approved by the Ethics Committee of the Department of Nursing, Physiotherapy and Medicine (approval code: EFM 488.25).

Authorization to conduct the study was also obtained from the management of the long-term care facility before participant recruitment. All participants received verbal and written information regarding the purpose of the study, the voluntary nature of participation, and their right to withdraw at any time without consequences for the care received. Written informed consent was obtained from each participant or, when applicable, from a family member or legal representative.

According to subsequent confirmation from the facilitating nurse, participants’ routine treatments, including psychotropic medications, remained stable during the four-week intervention; medication exposure itself was not systematically analyzed and stability was not prospectively monitored as a separate study outcome.

Participant confidentiality was protected through coded study identifiers. The analytical database was maintained separately from direct identifying information, and study data were stored on a password-protected and encrypted digital device accessible only to the principal investigator. Data processing was performed in accordance with the General Data Protection Regulation (EU) 2016/679 and the Spanish Organic Law 3/2018 on Personal Data Protection and Guarantee of Digital Rights [32,33].

## 3. Results

### 3.1. Participant Flow and Baseline Characteristics

**Figure 1:**
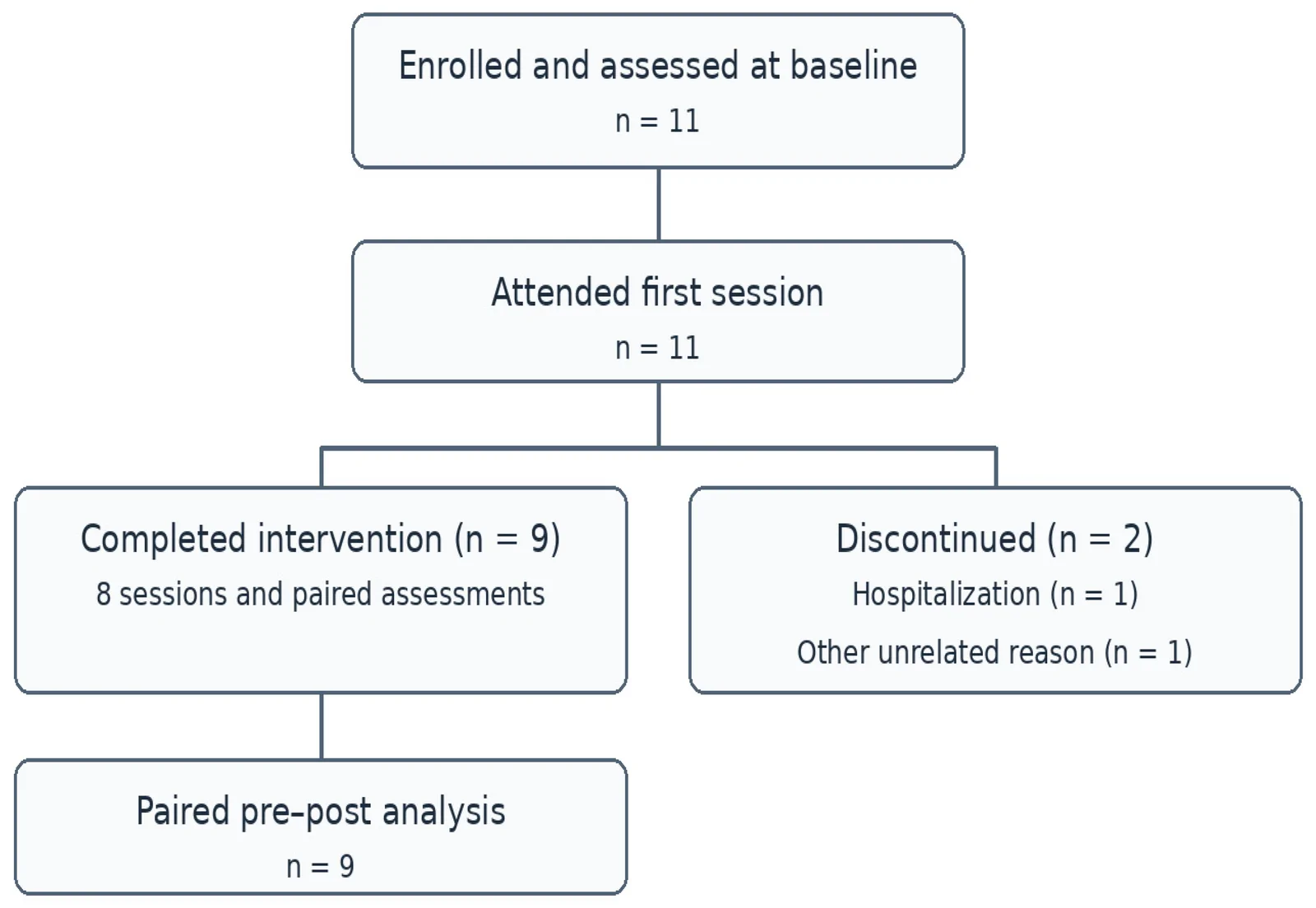
Participant flow during the four-week intervention.

Eleven residents were enrolled, of whom nine completed all eight sessions and both assessment time points (retention: 9/11; 81.8%). Two female participants aged 85 and 86 years attended the first session but discontinued before the second: one was hospitalized for a reason considered unrelated to the intervention and one discontinued for another reason considered unrelated to the intervention. Their baseline MoCA scores were 20 and 21, and GDS-15 scores were 7 and 6, respectively. The mean MoCA score among non-completers was 20.50, versus 22.89 among completers. These characteristics are described without inferential comparison owing to the very small number of withdrawals. Participant flow is presented in Figure 1.

Among the nine participants who completed the study, the mean age was 83.11 years (SD = 4.14), with a range of 78–89 years. Five participants were women (55.6%) and four were men (44.4%). The mean duration of residence in the long-term care facility was 53.33 months (SD = 46.52), ranging from 24 to 144 months. All participants included in the final analysis were classified as having mild cognitive impairment at baseline. Baseline characteristics of participants who completed the intervention are summarized in Table 1.

### 3.2. Feasibility and Acceptability

Of the 11 participants initially enrolled, nine completed the four-week program and the post-intervention assessment, corresponding to a retention rate of 81.8%. The nine participants who completed the study attended all eight scheduled sessions, corresponding to 72 of 72 expected participant-sessions (100%).

All eight intervention sessions were delivered as planned over the four-week period. Mean session duration was 39.63 ± 4.44 min, with sessions ranging from 33 to 45 min.

According to the facilitator’s session notes, all scheduled activities were delivered without reported modifications. No intervention-related incidents were reported; however, no standardized adverse-event surveillance procedure was implemented.

Acceptability among participants who completed the intervention was high. All nine participants rated their overall satisfaction with the program at the maximum score of 5 on the 5-point scale (mean = 5.00, SD = 0.00). In addition, all participants (9/9; 100%) indicated that they would be willing to participate in the program again.

### 3.3. Session-Based Engagement

Throughout the eight intervention sessions, participants showed moderate-to-high levels of behavioral engagement. Mean active participation was 3.65 ± 0.57 on the 5-point observational scale, with participant-level mean scores ranging from 2.88 to 4.38. Sustained attention showed the highest mean score (3.96 ± 0.44; range: 3.13–4.38), while mean social interaction was 3.89 ± 0.71 (range: 3.13–5.00).

These observational outcomes were analyzed descriptively, and no inferential comparisons were performed for the session-based engagement variables. Feasibility, acceptability, and session-based engagement outcomes are summarized in Table 2.

### 3.4. Exploratory Clinical Outcomes

Pre–post changes in cognitive function and depressive symptoms are presented in Table 3.

#### 3.4.1. Cognitive Function

MoCA scores increased from a mean of 22.89 ± 2.20 at baseline to 23.89 ± 2.03 after the intervention. The median score increased from 23.0 (IQR: 22.0–25.0) at baseline to 25.0 (IQR: 22.0–25.0) post-intervention. The median within-participant change was +1.0 point (IQR: 1.0–1.0).

The paired mean MoCA difference was +1.00 point (95% CI 0.46 to 1.54, t-based exploratory interval; Cohen’s dz = 1.41). Individual changes were 0, 0, +1, +1, +1, +1, +1, +2, +2; the 25th and 75th percentiles under linear interpolation were both +1.

Seven of the nine participants showed an increase in their MoCA scores, while two remained unchanged; no participant showed a decrease. The Wilcoxon signed-rank test indicated a statistically significant pre–post difference (exact two-sided *p* = 0.016).

Given the pilot design, small sample size, and absence of a control group, this finding should be interpreted cautiously and does not establish intervention efficacy.

#### 3.4.2. Depressive Symptoms

Mean GDS-15 scores decreased from 6.33 ± 1.00 at baseline to 6.00 ± 1.00 after the intervention. Median scores were 6.0 (IQR: 6.0–7.0) at baseline and 6.0 (IQR: 5.0–6.0) post-intervention.

The paired mean GDS-15 difference was −0.33 point (95% CI −0.72 to +0.05; Cohen’s dz = −0.67).

Three participants showed a one-point reduction in GDS-15 score, while six remained unchanged; no participant showed an increase in depressive symptom scores. The pre–post difference was not statistically significant (exact two-sided Wilcoxon *p* = 0.250).

No statistically significant change in depressive symptoms was observed over the four-week intervention period.

## 4. Discussion

This pilot study evaluated the feasibility, acceptability, and preliminary outcomes of a structured nurse-led active music program for older adults with cognitive impairment living in a long-term care facility. The main findings indicate that the intervention could be delivered within routine residential care, with an 81.8% retention rate and complete attendance among participants who completed the program. Acceptability was also high, as all participants reported the maximum satisfaction score and expressed willingness to participate again. In addition, participants demonstrated moderate-to-high levels of active participation, sustained attention, and social interaction throughout the sessions. Exploratory analyses showed a favorable pre–post change in cognitive screening performance, whereas depressive symptoms remained statistically unchanged.

The feasibility findings are particularly relevant because pilot studies are intended primarily to determine whether an intervention and its associated procedures can be successfully implemented before progressing to larger effectiveness trials [34]. In this study, the eight-session program was completed within the planned four-week period, with high attendance among participants and no reported safety incidents during the intervention. These findings are consistent with previous research suggesting that group music interventions can be feasible and acceptable in memory-care and long-term care environments [35]. However, implementation in these settings depends not only on the intervention itself but also on contextual factors such as staff involvement, organizational support, adaptation to residents’ needs, and compatibility with everyday care routines [18].

A distinctive feature of this intervention was its delivery by a registered nurse already integrated into the long-term care team. This may facilitate implementation because nurses have continuous contact with residents and detailed knowledge of their clinical condition, functional limitations, and individual preferences. Such familiarity may enable session intensity and activities to be adapted in real time while maintaining safety and continuity with the overall care plan. This approach is consistent with the broader need to develop non-pharmacological interventions that can be incorporated into existing care structures rather than relying exclusively on external professionals or additional services [10,17]. Nevertheless, the present findings should not be interpreted as demonstrating the superiority of nurse-led delivery over interventions conducted by other appropriately trained professionals.

All eight sessions were carried out during the nurse’s scheduled work hours, demonstrating that the program could be delivered within this facility’s routine care schedule.

Recent implementation studies of music-based interventions in long-term care facilities have highlighted the importance of staff availability, organizational support, and intervention fidelity [18,36].

The observational outcomes further support the acceptability of the intervention. Participants maintained moderate-to-high levels of active participation, sustained attention, and social interaction throughout the eight sessions. Group-based active music interventions may be particularly suitable for older adults living in long-term care facilities because they combine cognitive stimulation with opportunities for interpersonal communication and meaningful shared activity. Previous studies and systematic reviews have similarly described the potential of active music participation to enhance engagement, communication, and social connectedness among people with cognitive impairment or dementia [4,5,7,35]. These outcomes may be particularly relevant in long-term care settings, where cognitive decline frequently coexists with reduced social participation and limited opportunities for personally meaningful activities.

Regarding cognitive outcomes, MoCA scores showed a favorable short-term change following the intervention. Seven of the nine participants had higher post-intervention scores, while two remained unchanged. This finding is broadly consistent with previous evidence suggesting that music-based interventions may have beneficial effects on global cognition, memory, attention, and executive functioning in people with mild cognitive impairment or dementia [7,37,38]. However, the available evidence remains heterogeneous, and differences in intervention type, intensity, duration, participant characteristics, and outcome measures complicate direct comparisons across studies [8,37,38,39].

The observed cognitive change should therefore be interpreted as an exploratory finding warranting further investigation rather than as evidence of clinically meaningful intervention efficacy.

In contrast, no statistically significant change was observed in depressive symptoms as measured using the GDS-15. Although three participants showed a one-point reduction and none showed worsening, the overall pre–post difference was small. Previous systematic reviews have reported potentially favorable effects of music-based interventions on depressive, anxiety, and neuropsychiatric symptoms in people with cognitive impairment or dementia, although the magnitude and consistency of these effects vary considerably [8,38,39].

Importantly, the lack of measurable change in GDS-15 scores should be distinguished from intervention acceptability and engagement. Satisfaction, willingness to participate again, and behavioral participation capture different dimensions of the intervention than depressive symptom screening. The high levels of engagement observed during the sessions suggest that participants found the activities accessible and engaging, even though a measurable change in depressive symptoms was not detected over the study period.

The structured nursing framework used to plan the intervention may also contribute to its potential transferability to clinical practice. Integrating NANDA-I diagnoses, NIC interventions, and NOC-oriented outcomes provides a systematic way of linking identified care needs with specific nursing actions and expected dimensions of response [15,16]. In this study, these classifications were used as a clinical reasoning framework rather than as direct research outcome measures. This distinction is important because it allows standardized nursing terminology to support intervention planning while validated instruments are used to evaluate study outcomes.

From a healthcare-service perspective, these findings are relevant to the provision of care for a vulnerable population with complex cognitive and psychosocial needs. Long-term care facilities increasingly require interventions that are not only potentially beneficial but also feasible, acceptable, adaptable, and sustainable within routine practice. The present findings suggest that a structured active music program using readily available materials can be delivered by nursing staff within the care environment itself. If confirmed in larger studies, this model could provide an additional approach for incorporating structured non-pharmacological and person-centered activities into routine long-term care.

### 4.1. Limitations

This pilot study has several important methodological and contextual limitations that should be carefully considered when interpreting its findings. First, the single-group pre–post design and absence of a control group prevent causal attribution of the observed changes to the intervention. Factors such as repeated testing, nonspecific attention, temporal variation, or other concurrent aspects of care cannot be excluded. The one-point mean MoCA change is small in absolute terms and may reflect retest or practice effects or normal variability; neither the statistically significant paired result nor the standardized effect size establishes clinically meaningful intervention-related improvement.

The same nurse delivered the program, administered MoCA and orally administered GDS-15, and recorded engagement. Independent blinded assessment was not performed; assessor expectancy and observational biases cannot be excluded. No formal standardized fidelity checklist or external observer was used. No prospective feasibility thresholds were specified. The impact of delivering the intervention during scheduled nursing hours on workload, competing duties, staff well-being, and long-term organizational sustainability was not formally evaluated.

Second, the sample size was small, and participants were recruited using consecutive sampling in a single long-term care facility. These characteristics limit statistical power and restrict the generalizability of the findings. Nevertheless, small samples are consistent with the exploratory purpose of pilot and feasibility studies, whose primary objective is to evaluate implementation and generate information for the design of subsequent studies rather than to establish definitive effectiveness [34]. In addition, acceptability, engagement, and exploratory clinical outcomes were evaluated among participants who completed the intervention. Therefore, attrition may have introduced selection bias and may have resulted in an overestimation of acceptability and adherence.

Two non-completers had lower baseline MoCA scores than the average of completers. Since adherence, engagement, satisfaction, and willingness to repeat were measured only among completers, attrition may have overestimated these indicators relative to all enrolled residents. In addition, a single cultural and musical context limits transferability to other repertoires, populations, and care settings.

Third, the intervention lasted only four weeks, and no follow-up assessment was performed. It is therefore unknown whether the observed cognitive change would persist after completion of the program or whether a longer intervention would be required to influence more complex outcomes such as depressive symptoms or quality of life. The single-arm design also does not permit conclusions about optimal session duration or frequency, as alternative schedules were not compared.

Psychotropic exposure, prior music experiences, hearing differences, and additional staff attention were not systematically quantified or controlled. Although routine medicines reportedly remained unchanged according to the facilitator’s retrospective confirmation, medication-related confounding cannot be excluded. The small sample and limited variation in GDS-15 scores also constrained the ability to detect changes in depressive symptoms.

Fourth, session-based participation, attention, and social interaction were assessed using an observational instrument developed for the study. The five-point measure was not independently validated.

Although a 1–5 observational scale was used, its written behavioral anchors were not demonstrably established prospectively. Supplementary Material S3 documents the original response categories but does not reconstruct numerical behavioral anchors; the scale was not psychometrically validated.

Finally, acceptability was assessed using an ad hoc satisfaction questionnaire. Although this provided useful information for a feasibility study, future research should incorporate validated measures of acceptability, implementation outcomes, and participant experience.

### 4.2. Implications for Future Research

Future studies should evaluate the intervention using larger, multicenter samples and controlled designs, preferably randomized where feasible. Longer intervention and follow-up periods would help determine whether changes in cognitive performance are sustained over time and whether repeated exposure produces measurable effects on emotional well-being, social functioning, and quality of life.

Future multicenter studies should represent varied musical and cultural settings and compare session lengths and frequencies, account for cognitive heterogeneity, and assess perceived nurse workload, staffing, fidelity, adverse events, and costs. A priori progression criteria should be specified.

Future trials should also incorporate independent outcome assessors and validated measures of acceptability and implementation. Particular attention should be paid to intervention fidelity, organizational barriers and facilitators, staff training requirements, and the resources needed to integrate the program sustainably into routine long-term care [17,18].

Given the promising feasibility findings, subsequent research should also examine whether nurse-led delivery offers organizational or economic advantages compared with other models of music-based intervention. Such research would help determine whether this approach can be scaled across different long-term care environments while preserving person-centered adaptation and intervention quality.

## 5. Conclusions

This pilot study provides preliminary descriptive evidence regarding the feasibility and acceptability of a structured nurse-led active music program in a long-term care setting. Nine of the eleven enrolled residents completed the eight-session intervention, with high attendance, satisfaction, and willingness to participate again among completers.

A small exploratory pre–post increase in cognitive screening scores was observed, whereas depressive symptoms showed no statistically significant change. These findings should be interpreted cautiously and do not establish clinical efficacy.

Further research involving larger, controlled, and multicenter studies, with independent assessments and longer follow-up periods, is needed to confirm these preliminary findings and evaluate the potential integration of structured nurse-led active music programs into routine long-term care.

## Figures and Tables

**Table 1:** Baseline characteristics of all enrolled participants (*n* = 11), completers (*n* = 9), and non-completers (*n* = 2).

Characteristic	Enrolled (*n* = 11)	Completers (*n* = 9)	Non-Completers (*n* = 2)
Age, mean (SD), years	83.55 (3.83)	83.11 (4.14)	85.50 (0.71)
Women, *n* (%)	7/11 (63.6%)	5/9 (55.6%)	2/2 (100.0%)
Residence duration, mean (SD), months	47.64 (43.50)	53.33 (46.52)	22.00 (2.83)
Mild cognitive impairment, *n* (%)	11/11	9/9	2/2
MoCA, mean (SD)	22.45 (2.21)	22.89 (2.20)	20.50 (0.71)
GDS-15, mean (SD)	6.36 (0.92)	6.33 (1.00)	6.50 (0.71)

Note: SD = standard deviation; MoCA = Montreal Cognitive Assessment; GDS-15 = 15-item Geriatric Depression Scale.

**Table 2:** Feasibility, acceptability, and session-based engagement outcomes.

Outcome	Result
Retention, *n*/*N* (%)	9/11 (81.8)
Attendance among completers, attended/expected participant-sessions (%)	72/72 (100)
Session duration, min, mean ± SD	39.63 ± 4.44
Session duration, range (min)	33–45
Overall satisfaction (1–5), mean ± SD	5.00 ± 0.00
Willing to participate again, *n*/*N* (%)	9/9 (100)
Active participation (1–5), mean ± SD	3.65 ± 0.57
Active participation, range	2.88–4.38
Sustained attention (1–5), mean ± SD	3.96 ± 0.44
Sustained attention, range	3.13–4.38
Social interaction (1–5), mean ± SD	3.89 ± 0.71
Social interaction, range	3.13–5.00

Note: Session-based engagement values represent the distribution of each participant’s mean score across the eight intervention sessions. SD = standard deviation.

**Table 3:** Exploratory pre–post clinical outcomes among participants who completed the intervention (*n* = 9).

Outcome	Baseline, Mean ± SD	Post-Intervention, Mean ± SD	Baseline, Median (IQR)	Post-Intervention, Median (IQR)	Median Change (IQR)	Exact *p*-Value
MoCA	22.89 ± 2.20	23.89 ± 2.03	23.0 (22.0–25.0)	25.0 (22.0–25.0)	+1.0 (1.0–1.0)	0.016
GDS-15	6.33 ± 1.00	6.00 ± 1.00	6.0 (6.0–7.0)	6.0 (5.0–6.0)	0.0 (−1.0–0.0)	0.250

Note: MoCA = Montreal Cognitive Assessment; GDS-15 = 15-item Geriatric Depression Scale; SD = standard deviation; IQR = interquartile range. Higher MoCA scores indicate better cognitive performance, whereas higher GDS-15 scores indicate greater depressive symptom burden. Pre–post comparisons were performed using the Wilcoxon signed-rank test with two-sided exact *p*-values.

## Data Availability

The data supporting the findings of this study are available from the corresponding author upon reasonable request. The data are not publicly available due to privacy and ethical considerations.

## Supplementary Materials

The following supporting information can be downloaded at: https://www.mdpi.com/article/10.3390/healthcare14213732/s1, Supplementary Material S1: Structured nurse-led active music intervention protocol; Supplementary Material S2: Study-specific acceptability assessment; Supplementary Material S3: Session-based observational recording form.
