# Supplementary material for: Feasibility and Acceptability of a Nurse-Led Active Music Program for Older Adults with Cognitive Impairment in Long-Term Care: A Pilot Quasi-Experimental Study
Source: Healthcare (Basel). 2026 Nov 3;14(21):3732. doi: 10.3390/healthcare14213732 (PMC13762540; doi:10.3390/healthcare14213732)
Supplement: Supplementary file 1 [file healthcare-14-03732-s001.zip › healthcare-4591098-supplementary.pdf]

Supplementary Materials

S1. Structured Nurse-Led Active Music Intervention Protocol

The eight-session intervention protocol used in the study is summarized below. The protocol was established in writing before the intervention began.

Design: eight group sessions, twice weekly for four weeks. Each follows three phases: (1) opening temporal orientation and guided diaphragmatic breathing, with gentle arm movement; (2) main session-specific musical activities combining singing, rhythm, movement, cognition and group interaction; (3) closing relaxation with soft instrumental music, optional recollection and expression of feelings, and positive reinforcement.

Session 1. Familiarization and therapeutic rapport

Familiar songs such as "Clavelitos"; guided group singing, clapping to the pulse, and gentle coordinated arm movements.

Session 2. Autobiographical memory

Recognizing songs such as "La chica ye-yé" and "Ojos verdes"; completing lyrics, recollection of life events, ordering lyric fragments, claps and finger snaps.

Session 3. Language activation

Call-and-response singing, such as "Guantanamera"; lyric completion, word generation, simple verbal improvisation, and tambourine accompaniment.

Session 4. Motor coordination

Seated clapping-knee sequences, alternating arms, wrist turns, leg lifts to familiar rhythms; maracas and tambourines.

Session 5. Sustained attention

Following tempo changes in familiar songs, music-stop activities, fragment recognition, and imitation of progressive rhythmic sequences.

Session 6. Social interaction

Group chain singing, musical bingo, synchronized percussion, and participant-led group rhythm with songs such as "Que viva España".

Session 7. Emotional regulation

Listening to excerpts, labeling emotions, personal music-emotion associations, gentle seated rocking and slow rhythmic breathing.

Session 8. Integration and closure

Recalling songs from previous sessions, combining movement, singing and rhythm, participants choosing songs and repeating preferred activities.

Provider: a facility-employed registered nurse without specialist music therapy qualification. The written protocol existed before delivery. Qualitative session records were maintained; no independent or standardized prospective fidelity checklist was used.

S2. Study-Specific Acceptability Assessment

The available study records document overall satisfaction on a five-point scale (1 = completely dissatisfied; 5 = completely satisfied) and willingness to repeat the intervention (yes/no). Both measures were collected among the nine intervention completers. The original questionnaire is no longer available; the information below describes the recorded items and response coding rather than reproducing the original questionnaire.

Item	Response options	Variable
Overall satisfaction	1–5 Likert scale	Satisfaction_1to5
Willingness to participate again	No / Yes	Willingness_to_repeat (0 = No; 1 = Yes)
Additional comments (if any)	Optional free text	Final_comments

S3. Session-Based Observational Recording Form

The study-specific session record included participant and session details (session number, date, duration, attendance and reasons for absence); six five-point observational indicators (general participation, sustained attention, response to rhythm, engagement in musical activities, verbal expression and social interaction); and qualitative sections for emotional, cognitive and social responses, activities delivered, incidents and suggested adjustments. The three dimensions analyzed in the manuscript were participation, attention and social interaction.

Scoring limitations

The study's three analyzed dimensions were active participation, sustained attention and social interaction. The original form used 1–5 boxes but did not supply fully documented prospective numerical behavioral anchors for each value. No numerical behavioral anchors have been reconstructed in this supplement; retrospective interpretations cannot be presented as prospectively established criteria. The same nurse completed observations within 15 minutes after sessions; no independent rater or inter-rater reliability assessment was used.

S3 (continued). Original Recording Form – Complete Field Structure

The following is a structured English transcription of the fields and response options of the original session record. It is not a facsimile and does not reproduce completed participant forms. No scoring anchors are reconstructed.

Participant identification

Field	Response
Participant code	As recorded
Age	As recorded
Sex	As recorded
Diagnosis / cognitive impairment level	As recorded
Baseline MoCA-B	As recorded

Baseline Mini-Cog	As recorded
Baseline GDS-5/15	As recorded
Start date	As recorded

Session information

Field	Response
Session number (1–8)	As recorded
Date	As recorded
Duration (minutes)	As recorded
Attendance (Yes/No)	As recorded
Reason for nonattendance	As recorded

Five-point observations

Field	Response
General participation	1 2 3 4 5
Sustained attention	1 2 3 4 5
Response to rhythm	1 2 3 4 5
Musical activities	1 2 3 4 5
Verbal expression	1 2 3 4 5
Social interaction	1 2 3 4 5

Emotional response (Yes/No/Comments)

Field	Response
Spontaneous smile	Yes / No / Comments
Bodily relaxation	Yes / No / Comments
Positive comments	Yes / No / Comments
Intense emotion	Yes / No / Comments
Negative comments	Yes / No / Comments

Cognitive response (Yes/No/Comments)

Field	Response
Temporal orientation	Yes / No / Comments
Recalls melodies	Yes / No / Comments

Completes song verses	Yes / No / Comments
Follows instructions	Yes / No / Comments
Musical reminiscence	Yes / No / Comments

Social response (Yes/No/Comments)

Field	Response
Greets peers	Yes / No / Comments
Eye contact	Yes / No / Comments
Spontaneous interaction	Yes / No / Comments
Group coordination	Yes / No / Comments
Conversation before/after	Yes / No / Comments

Intervention activities (check)

Field	Response
NIC 4400 Group singing	As recorded
NIC 4400 Rhythm/percussion	As recorded
NIC 4400 Rhythmic movement	As recorded
NIC 4400 Musical memory	As recorded
NIC 4720 Guided attention	As recorded
NIC 4720 Musical recall	As recorded
NIC 4860 Memory evocation	As recorded
NIC 4920 Emotional validation	As recorded

Additional record fields

Field	Response
Incidents / notes	As recorded
General session impression	As recorded
Future adaptation required (Yes/No)	As recorded
Suggested adjustments	As recorded

Only general participation, sustained attention and social interaction were included in the three principal quantitative outcomes. The form also includes optional fields for measures that were not necessarily administered; their presence does not establish that those measures were collected.
